# Biomechanical Evaluation of Dual Plate Configurations for Femoral Shaft Fracture Fixation

**DOI:** 10.1155/2019/5958631

**Published:** 2019-04-28

**Authors:** Marc El Beaino, Randal P. Morris, Ronald W. Lindsey, Zbigniew Gugala

**Affiliations:** Department of Orthopaedic Surgery and Rehabilitation, The University of Texas Medical Branch, Galveston, TX, USA

## Abstract

**Aim:**

This study aimed at comparing the mechanical properties of conventional and locking dual plates in adjacent and orthogonal orientations for the surgical fixation of transverse femoral shaft fractures. It also assessed the failure mechanics after dual adjacent and orthogonal locking plate removal.

**Methods:**

Thirty-two composite femurs were transversally osteotomized and randomly assigned for fixation with either dual locking or compression plates in an adjacent or orthogonal configuration. Sixteen specimens were preloaded axially to 20 N and single-leg stance loads were simulated. The remaining sixteen constructs were subjected to torsional loads of 10 Nm at a rate of 10 Nm/s in external and internal rotation of the femoral head in relation to the knee. Overall combined rotational stiffness was calculated. Eight different specimens with no osteotomy underwent the same experiments after dual locked plate removal and were tested to failure in combined eccentric axial and torsional modes. Data were statistically processed using a two-tailed* t*-test and one-way analysis of variance for the comparison of means between two or more groups, respectively.

**Results:**

Orthogonal constructs were statistically stiffer in axial loading compared to their adjacent counterparts in both conventional and locking configurations (p<0.001). Dual locking plates provided higher torsional stiffness than conventional ones within each plate orientation (p<0.01). Neither axial/torsional strength nor failure loads differed between constructs that had adjacent or orthogonal dual locking plates instrumented and then removed (p>0.05).

**Conclusions:**

In both orthogonal and adjacent orientations, double locking plates provide higher stability than their dual conventional counterparts. Orthogonal dual plate configuration is more stable and biomechanically superior to dual adjacent plating for constructs fixed with either standard compression or locking plates.

## 1. Introduction

Femoral shaft fractures are common lower-extremity insults that typically result from high-energy injuries [1]. They are associated with varying degrees of instability and are of special concern, particularly in the elderly population [2, 3]. They can usually be effectively treated with placement of an intramedullary nail, which exhibits sufficient biomechanical axial stability that translates clinically in high union and low infection rates [4–6]. However, when associated with a high degree of comminution, nonunion, open growth plates, poor bone quality, and/or extensive contamination, femoral diaphyseal fractures may not be manageable with this technique, and plate osteosynthesis might be required for better surgical reduction [7, 8]. In such context, biomechanical axial and rotational loading or bending analyses have shown plate-screw fixation to provide comparable or higher long-bone construct stiffness compared to intramedullary nail [9–11].

Compression and locking plates utilize different mechanisms to attain diaphyseal fracture reduction and healing  [12]. The former achieves stabilization at the bone-plate interface through compression of the plate against the underlying bone on both sides of the fracture, whereas the latter creates a unified construct consisting of threaded screw-heads locked into the plate without compressing the bone underneath. Additionally, the quality of fixation of long-bone shaft fractures has been shown to vary depending on the plate length used [12]. In surgical reduction of distal femoral fractures, shorter plate length (<9 holes) correlated with higher implant breakage rates [13]. Conversely, keeping the plate hole adjacent to the fracture unfilled with a screw extended the time interval for implant failure [14].

Single-plate osteosynthesis may be ineffective in achieving adequate reduction of fractures subjected to increased loads, a finding that led some authors to recommend dual plating in such circumstances [9, 10, 15]. Although dual orthogonal plating was shown to be biomechanically and clinically superior to single locked plating in the management of femoral midshaft fractures, there are some technical difficulties associated with this technique, such as the risk of extensive soft-tissue stripping and periosteal circulation disruption [16, 17]. Placing dual plates across a fracture in an adjacent orientation may constitute an alternative fixation requiring less surgical exposure, while possibly providing similar biomechanical stability to orthogonally configured plates. However, the biomechanical merits of this surgical modality are yet to be determined in comparison to the dual plating orthogonal orientation.

The objectives of this study were to compare the mechanical properties of conventional and locking dual plates in an adjacent or orthogonal orientation in the surgical reduction and fixation of femoral diaphyseal fractures. This investigation also assessed the strength and failure mechanics of the femur after dual adjacent and orthogonal locking plate removal.

## 2. Methods

### 2.1. Ethical Statement

This biomechanical project involved neither live animals nor human participants and did not require Institutional Review Board approval prior to its completion.

### 2.2. Fracture Model and Construct Instrumentation

**Figure 1:**
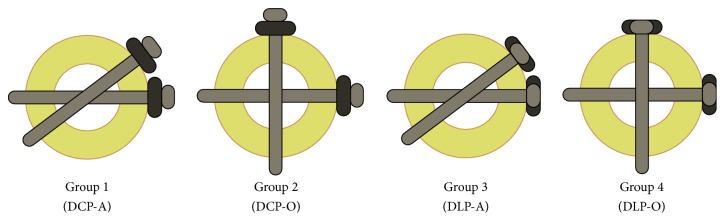
Transverse diagrams representing the 4 tested constructs. The specimens were randomly assigned to 4 distinct groups: Group 1 (DCP-A) consisted of dual dynamic compression plates in an adjacent orientation; Group 2 (DCP-O) included orthogonal dual compression plate constructs; Group 3 (DLP-A) encompassed adjacent dual locking plates; and Group 4 (DLP-O) contained dual locking constructs in an orthogonal configuration.

Thirty-two composite third-generation medium left femurs (Model 3303, Pacific Research Laboratories, Sawbones Worldwide, Vashon, WA) were randomly assigned for fixation with 1 of 4 distinct plate configurations (Figure 1).

A diaphyseal femoral midshaft fracture was simulated by performing a transverse osteotomy. The fracture was spanned and stabilized with either 10-hole 4.5 mm stainless steel dynamic dual compression plates (DCP) (DePuy Synthes Inc., West Chester, PA) or dual locking plates (DLP) (Smith & Nephew Inc., Memphis, TN). Axial and torsional analyses included 16 identical specimens for each mode, with 4 sets of 4 composite femurs (Table 1).

Plates were placed 8.5 cm distal to the tip of the greater trochanter along the lateral side of the femur. Pilot holes, 3.5 mm in dimension, were drilled into each of the outermost 4 holes on the proximal and distal sides of each plate. Eight bicortical 4.5 mm screws per plate were then inserted with a torque wrench into each of the pilot holes, securing the plate to the synthetic bone. After instrumentation of the lateral plate, a hacksaw was used to create a 1 cm osteotomy gap in the middiaphysis of the femur and center of each 10-hole plate so that the closest holes to the defect could be left empty as per the surgical protocol [14, 18]. The second plate was then instrumented on the femur. In 8 constructs, an adjacent 10-hole DCP (4 specimens) or DLP (4 specimens) was placed immediately anterior to the respective lateral DCP or DLP, with approximately 2 mm of space between each plate. This second plate was oriented at the same level of the first plate, with the 4.5 mm screws inserted obliquely and directed away from the defect (adjacent configuration, DCP-A or DLP-A). The remaining 8 femurs were instrumented in a similar fashion, with the second DCP or DLP oriented at 90 degrees from the first plate, but on the anterior aspect of the femur (orthogonal configuration, DCP-O or DLP-O).

To assess the strength and failure mechanics of the femur after locking plate removal, the second experimental arm consisted first of instrumenting dual locking plates in either an adjacent or an orthogonal orientation (4 synthetic femurs in each group). In both configurations, the plates were centered in a similar fashion as described above, not spanning any diaphyseal femoral midshaft osteotomy. The screws and plates were then removed. A total of 8 synthetic constructs, each containing 16 holes (8 holes per plate) in an adjacent (4 specimens) or orthogonal (4 specimens) orientation, were therefore generated.

### 2.3. Biomechanical Setup and Testing

**Figure 2:**
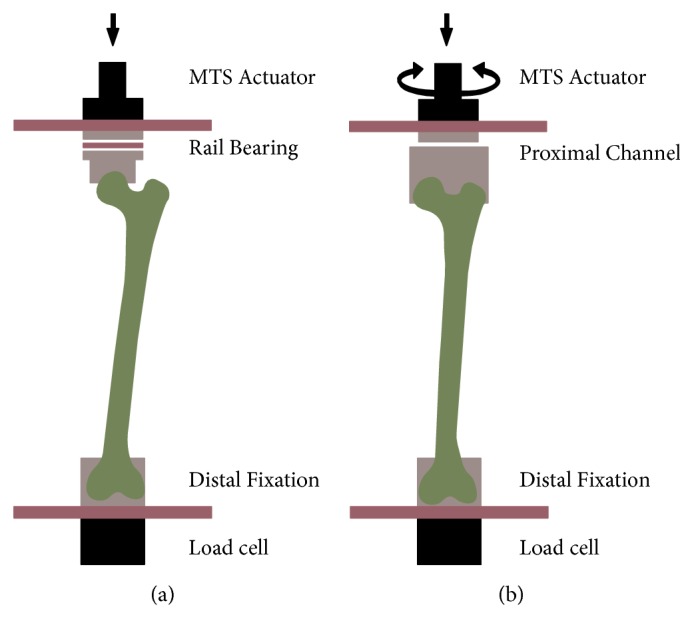
Construct mounting on the MTS testing machine. All constructs were distally potted in PMMA-filled aluminum box in a 10-degree eccentric adduction. Mechanical testing was performed in (a) axial and (b) torsion loadings.

For axial loading (20 constructs, 16 with plates and 4 without), the condyles and distal ends of each synthetic femur were potted with polymethylmethacrylate (PMMA) in an aluminum box in 10 degrees of adduction relative to the anatomical femoral axis. Femurs were rigidly secured distally to the load cell of a servohydraulic materials testing machine (858 Mini-Bionix, MTS Systems Corporation, Eden Prairie, MN) in a custom loading frame that allowed force transfer along the biomechanical axis of the femur (Figure 2). Axial load was applied to the femoral head through a concave spherical cup and a rail bearing. The constructs were preloaded to 20 N, and then 678 N was applied for 10 loading cycles at a rate of 0.5 Hz to simulate body-weight loads. Load displacement curves were generated, and stiffness was calculated as the slope of the 10-cycle loading curve.

For torsional loading (20 constructs, 16 with plates and 4 without), the composite femurs were potted distally as before, while the head and greater trochanter were set within a custom aluminum plate channel that would allow rotational forces to be applied proximally. Each construct was loaded to 10 Nm at a rate of 10 Nm/s in both external and internal rotation of the head in relation to the knee (Figure 2). Load displacement curves were generated and stiffness was calculated as the slope of the 10-cycle loading curve.

After being subjected to the biomechanical testing protocol described above to determine axial and torsional stiffness, the eight 16-hole constructs with no plates were then tested in combined eccentric axial compression and torsion until failure. Peak combined axial and rotational failure loads were recorded and analyzed.

### 2.4. Statistical Analysis

Data were normally distributed, justifying the use of parametric statistical tests. Comparisons of axial and torsional stiffness between the different plate configurations and types were computed with a one-way analysis of variance (ANOVA) and a post hoc Scheffé test for intergroup analysis. Comparisons of axial/rotational stiffness and failure torque between adjacent and orthogonal constructs with the plates removed were computed with a two-tailed Student's* t*-test. Post hoc analysis was also conducted to assess the power of the study. All analyses were performed using SPSS® version 24 (IBM Corp., Armonk, New York, USA).

## 3. Results

The instrumentation and subsequent testing were completed according to the protocol described. All constructs were successfully tested as intended with no outliers.  Table 2 summarizes the axial and torsional stiffness for all plate configurations.

**Figure 3:**
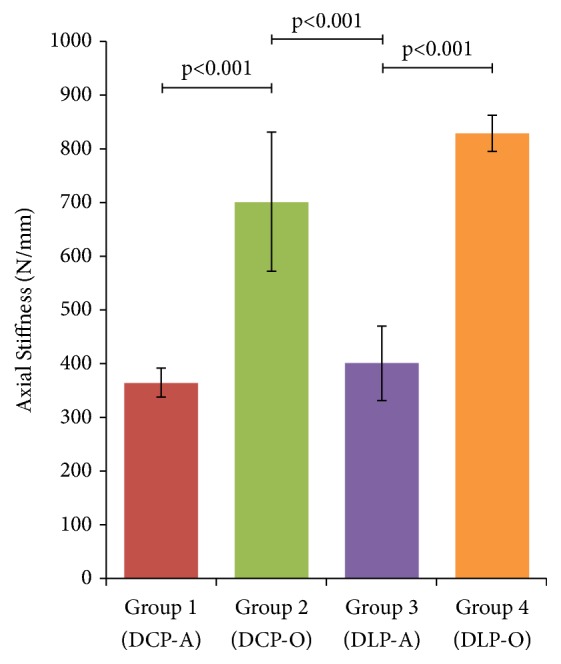
Analysis of the axial stiffness of the different constructs. DCP-A and DLP-A had significantly lower axial stiffness compared to their respective DCP-O and DLP-O constructs (p<0.001). DCP-O plating was more rigid axially compared to DLP-A (p<0.001), but not to DLP-O (p=0.19) specimens.

In axial loading, orthogonal plate configurations provided significantly superior compressive stiffness than adjacent orientations in both conventional and locking-plate subgroups. Group 1 (DCP-A) exhibited an axial mean stiffness of 364.9 N/mm (95% CI 311.2–418.5 N/mm), compared to 702.1 N/mm (95% CI 443–961.2 N/mm) in Group 2 (DCP-O) (p<0.001) (Figure 3). Similarly, DLP-O (Group 4) and DLP-A (Group 3) yielded axial stiffness averages of 829 N/mm (95% CI 761.7–896.4 N/mm) and 401 N/mm (95% CI 262.6–539.3 N/mm), respectively (p<0.001). Plate types did not affect the bending stiffness of either adjacent (DCP-A versus DLP-A, p=0.93) or orthogonal (DCP-O versus DLP-O, p=0.19) constructs. Interestingly, the rigidity of dual orthogonal compression (DCP-O) plates was superior to the adjacent locking (DLP-A) constructs (p<0.001).

**Figure 4:**
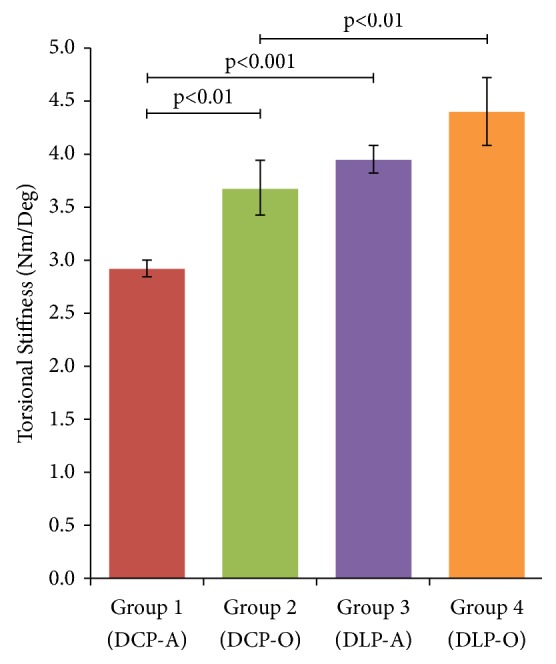
Rotational stiffness testing of the different plating techniques. DLP-A plates were statistically stiffer in torsion than DCP-A constructs (p<0.001). Similarly, DLP-O constructs exhibited higher rotation rigidity than DCP-O plating (p<0.01). Although DCP-A plates were less rigid than DLP-A (p<0.001), the difference did not hold for their orthogonal counterparts (DCP-O versus DLP-O, p=0.09).

In torsional testing, DCP-O constructs (Group 2) exhibited higher stiffness compared to their adjacent DCP-A counterparts (Group 1), with mean values of 3.7 Nm/deg (95% CI 3.1–4.2 Nm/deg) and 2.9 Nm/deg (95% CI 2.8–3.1 Nm/deg), respectively (p<0.01) (Figure 4). Group 3 (DLP-A) constructs exhibited a rotational stiffness mean of 4 Nm/deg (95% CI 3.7–4.3 Nm/deg), compared to 4.4 Nm/deg (95% CI 3.8–5.1 Nm/deg) in Group 4 (DLP-O) specimens, but the difference did not reach statistical significance (p=0.09). Dual locking plating constructs (DLP-A and DLP-O) were statistically stiffer than conventional plating constructs (DCP-A and DCP-O) within each plate orientation (p<0.01). The rigidity of the DCP-O plating was not superior to the one exhibited by the DLP-A constructs (p=0.41).

Dual locking plate removal demonstrated no statistical differences in axial and torsional stiffness among the resulting constructs. Adjacent holes specimens showed a mean axial stiffness of 1100 N/mm (95% CI 1011.5–1190.1 N/mm) compared to 1136.8 N/mm (95% CI 1075.6–1197.9 N/mm) for constructs with orthogonal holes (p=0.53). Similarly, specimens with adjacent holes exhibited a mean torsional stiffness of 7.7 Nm/deg (95% CI 7.4–8 Nm/deg) compared to 7.4 Nm/deg (95% CI 7–7.7 Nm/deg) for constructs initially instrumented with plates in an orthogonal orientation (p=0.2). Orthogonally configured constructs failed at an average of 5056.2 N (95% CI 4315.1–5797.3 N) axially at a 25 Nm (95% CI 21.1–29 Nm) of torque, whereas their adjacently oriented counterparts broke at an axial force mean of 4764.3 N (95% CI 4249.3–5279.2 N) and a torque of 23.9 Nm (95% CI 21.1–26.5 Nm) (p=0.55 and p=0.65, respectively).

## 4. Discussion

Femoral shaft fractures are of special concern in elderly individuals, who are prone to osteoporosis and other associated comorbidities. These common traumatic injuries are most frequently treated with open internal surgical reduction, which aims at providing a stiff and rigid construct while allowing early patient mobility [19–21]. Fixation may be achieved by femoral intramedullary nailing or plates/screws osteosynthesis. The former technique is considered the benchmark therapeutic modality in most cases, but its use is limited by inherent risks, such as femoral head necrosis and leg length discrepancy [6, 22, 23]. Moreover, it may not be always considered the appropriate or recommended surgical procedure such as in juxta-articular or comminuted fractures, revisions of failed femoral unions, and injuries associated with vascular disruption, femoral canal obstruction, or pronounced contamination [7, 8]. In such circumstances, biomechanical analyses have reported sufficient axial and torsional stabilities with plates/screws fixation techniques [17, 24–27].

Dual plating is a surgical fixation method that has been mainly used for the management of distal and proximal humeral fractures [11, 28–31]. While exhibiting higher stiffness in axial bending and torsion, this procedure has been limited by a high rate of infection, owing to the increased amount of soft-tissue stripping and periosteal vascular compromise associated with it. However, on multiple occasions, it may become the last operative alternative in the surgeon's armamentarium for the treatment of comminuted and/or complex fractures. Biomechanically, humeral shaft fractures managed with dual locking plates in an orthogonal orientation outperformed adjacent constructs in axial and rotational analyses [32]. This surgical technique yielded good functional outcomes in 11 of 16 (68.8%) patients with comminuted distal femoral fracture at one-year follow-up, with one (6.3%) and two (12.5%) individual(s) exhibiting plate failure and infection, respectively [33]. Cheng et al. compared dual orthogonal locked plating to interlocking intramedullary nailing in the management of femoral diaphyseal fractures and showed no differences in the rates of complications or bone union [17]. They recommended locked double plating in polytraumatic patients, in whom an interlocking cephalomedullary nail construct is deemed inappropriate. Similarly, both dual plating and exchange intramedullary nailing combined with plate augmentation achieved comparable high rates of unions in patients with femoral diaphyseal nonunion [34].

A retrospective study investigated locked implants in staged dual plating of traumatic open supracondylar femoral fractures and revealed a 100% union rate in all 15 patients analyzed [35]. This finding was corroborated by a more recent clinical report, which utilized minimally invasive simultaneous dual plate osteosynthesis approach with staged bone grafting for the surgical reduction of distal femoral fractures [36]. Dual plating fixation achieved an adequate quality of supracondylar femoral fracture reduction with no implant loosening during follow-up [37]. While comparing conventional to locking dual plating constructs, Jazrawi et al. noted that the latter model was associated with higher stiffness in bending testing in the surgical reduction of distal femoral fractures [27]. A recent study documented good functional outcomes in 14 (93%) of 15 patients who presented with femoral diaphyseal fracture and chest injury and underwent the orthogonal dual plating fixation technique [38]. However, these studies did not compare the biomechanical properties of the adjacent versus orthogonal dual plate configurations, an important variable to account for in the patient's evaluation and management [39].

In axial compression, our results indicate that dual orthogonal plating constructs were stiffer compared to dual adjacent plating fixations. Orthogonal dual compression constructs exhibited higher stiffness compared to dual adjacent locking constructs, which highlights the relevance of the plate orientation in reducing femoral shaft fractures. In rotational load testing, both locking and compression orthogonal constructs had higher rigidity compared to their respective parallel counterparts, but this difference reached statistical significance in the conventional compression plate subgroup only. Taken together, our data suggest that orthogonal plating yields higher biomechanical stiffness than adjacent constructs, with dual locking orthogonal plating outperforming all other surgical fixation techniques. Our findings are in disagreement with the results of Jazrawi et al., who documented that locking plating had similar fixation stability compared to their dynamic compression counterparts in axial loading [27]. These discrepancies might be due to the divergent properties of synthetic composite femurs used in our analysis compared to embalmed cadaveric human bones in their biomechanical study. Other explanations that may account for such discordances may be inherent to the difference in the fracture pattern, plate types, or configurations tested in both analyses.

Dual locked plate removal did not yield any difference in axial and torsion loading between orthogonally and adjacently configured composite femurs. Similarly, peak combined axial and rotational failure loads were comparable between both groups. These interesting findings were opposite to our initial expectation, in which we postulated that the relatively smaller distance between adjacent screw holes might induce a higher local stress raiser that requires lower energy to fail, compared to the longer distance created in orthogonally oriented constructs. The failure risk being associated more with the screw diameter or number, rather than their location, might explain our results.

Our study is subject to some limitations. While the sample size of composite femurs may have appeared relatively small, all biomechanical metrics analyzed to address our primary hypothesis were well powered. A post hoc analysis revealed 98% to 100% power in almost all measures. The only underpowered evaluation (74%) was the torsional load stiffness comparison between orthogonal and adjacent dual locking plates. It is possible that we might have been able to identify a statistically significant difference between these construct groups with a larger sample. Moreover, composite femurs may not reflect the bone biology of the osteopenic elderly patient population, and the testing methodology did not simulate all musculotendinous forces on the femur that would be found in vivo. The second experimental arm aimed at analyzing fractured femurs that have completely healed. This might be subjected to some drawbacks, in that the fracture site might still be fragile and the bone quality not totally restored. However, this design allowed us to study a homogenous biomechanically human-equivalent and validated synthetic femoral model without the need to account for interspecimen volumetric variations or assess the bone mineral density, a confounding variable commonly encountered with human cadaveric studies [40]. Additionally, our choice of 10-hole plates could be justified by the recommendation of having at least 8 to 10 screws purchased by the plate-bone interface on both the proximal and distal femoral shaft fragments in order for the plate to absorb the applied load [41].

In summary, orthogonal and adjacent dual plating are appropriate operative techniques for femoral shaft fracture fixation. Locking plates allow higher construct rigidity and stability compared to conventional dynamic compression plates. Clinical studies with comparison of both surgical modalities are needed to analyze the potential complications' incidence and functional outcomes.

## Figures and Tables

**Table 1:** The 4 plate configurations tested in the axial/torsional biomechanical modes.

Plates	Group	Plate Configuration	Sample Size (N)
Compression	1 (DCP-A)	Adjacent dual dynamic compression plates	4
	2 (DCP-O)	Orthogonal dual dynamic compression plates	4

Locking	3 (DLP-A)	Adjacent dual locking plates	4
	4 (DLP-O)	Orthogonal dual locking plates	4

**Table 2:** Mean axial and torsional stiffness of the 4 plating constructs.

Group	Axial Stiffness Mean (N/mm)	Torsional Stiffness Mean (Nm/deg)
DCP-A (Group 1)	364.9 (95% CI 311.2 – 418.5)	2.9 (95% CI 2.8 – 3.1)
DCP-O (Group 2)	702.1 (95% CI 443 – 961.2)	3.7 (95% CI 3.1 – 4.2)
DLP-A (Group 3)	401 (95% CI 262.6 – 539.3)	4 (95% CI 3.7 – 4.3)
DLP-O (Group 4)	829 (95% CI 761.7 – 896.4)	4.4 (95% CI 3.8 – 5.1)

## Data Availability

The data used to support the findings of this study are available from the corresponding author upon request.
